# Survey dataset on student perceptions and experiences of quality assurance in Vietnamese universities

**DOI:** 10.1016/j.dib.2023.109305

**Published:** 2023-06-09

**Authors:** Hien Thi Thu Ta, Cuong Huu Nguyen, Hung Thai Le, Nhung Thi Tuyet Pham, Huong Thi Pham, Nhung Thi Trinh

**Affiliations:** a VNU University of Education, Vietnam National University, Hanoi, Viet Nam; b Center for Education Accreditation, Vietnam National University, Hanoi, Viet Nam; c Department of Quality Assurance, Van Lang University, Ho Chi Minh City, Viet Nam; d Education Research Group, Van Lang University, Ho Chi Minh City, Viet Nam; e College of Foreign Languages, Hue University, Hue City, Viet Nam; f Department of Educational Sciences, Ho Chi Minh City University of Education, Ho Chi Minh City, Viet Nam

**Keywords:** Quality management, Accreditation, Higher education, Developing country, Internal quality assurance, Assessment, Evaluation, Student engagement

## Abstract

This paper presents a dataset from a survey of student perceptions and experiences of quality assurance in Vietnamese higher education institutions. Data were collected from July to September 2020 using the online survey via Google Forms. The survey was sent to students via their email and social media, and there were 1323 valid responses. The data collection instrument was developed based on an international survey administered by UNESCO. The survey was designed to elicit data with respect to students’ views on institutional quality policy and model, quality assurance procedures and tools, and student survey. The dataset serves as an insightful reference for institutional practitioners and policymakers in quality assurance to revise internal quality assurance policies and instruments to enhance the quality of teaching and learning. Moreover, the dataset could be of interest to other educational researchers who can use it to investigate students’ understanding and viewpoints on quality assurance.

**Specifications Table**

**Table:** 

Subject	Education
Specific subject area	Quality assurance
Type of data	Table
How the data were acquired	Targeted online survey
Data format	Raw
Description of data collection	The data was collected through a Vietnamese questionnaire consisting of 60 closed-ended questions. The questionnaire was administered in five major cities in different regions of Vietnam via Google Forms. After three months (July – September 2020), the survey obtained 1323 valid responses, most of which were from undergraduate students.
Data source location	Institution: Van Lang University City/Town/Region: Ho Chi Minh City Country: Vietnam
Data accessibility	Repository name: Mendeley Data Data identification number: 10.17632/k4cbg4fz6t.2 Direct URL to data: https://data.mendeley.com/datasets/k4cbg4fz6t/2

## Value of the Data

• The data enable researchers interested in higher education quality assurance to gain insights of students’ perceptions and experiences on quality assurance policy and implementation as well as verify the research's findings.
• The dataset offers valuable resources for policymakers, institutional leaders, educational managers and quality assurance specialists to make policies on quality enhancement for their programmes and institutions.
• The dataset helps contribute to knowledge management related to quality assurance procedures and instruments from students’ viewpoints.

## 1 Data Description

Quality assurance plays an important part in everyday activities and development strategies of all the higher education institutions. To ensure and enhance the quality of teaching, learning, research and management, universities collect feedback from stakeholders including students, lecturers, support staff, alumni and employers. They have substantial influence in the effectiveness of the university's functioning by participating in decision-making units and procedure related to quality assurance [1,2]. Among these stakeholders, students are considered the most influential internal stakeholders [3]. They have been perceived as more and more significant in the legitimacy of quality assurance processes. Specifically, they are involved in evaluations and in revisions of the learning outcomes as well as providing critical feedback on academic and non-academic support services [1,4]. Moreover, students can participate in quality assurance activities by giving feedback on the courses or programmes they have taken, helping to develop the curricula, participating in institution decision-making processes, or by speaking on behalf of students in a variety of ways, such as through a student union or other representative bodies [5].

In Vietnam, the national education system has been influenced by China, France, the Soviet Union and the United States [6]. In the 2019-2020 academic year, Vietnam had 237 universities with over 1.7 million students [7]. Quality assurance was officially introduced to the higher education sector in 2003. Initial achievements of quality assurance implementation (for example, institutional and programme accreditation, the development of the internal quality assurance system) have been observed in many universities. Particularly, student engagement in quality assurance processes has also been reported [8,9]. However, their role is only limited to the evaluation of courses, the most popular activity in most of the Vietnamese universities [10]. A survey containing 60 close-ended questions were developed to convey Vietnamese students’ perceptions and experiences on quality assurance in their universities. After three months of administration via Google Forms, the survey obtained 1323 valid responses. The associated dataset is presented in 11 tables below. Additionally, demographic characteristics of the respondents are presented in Table 12. Moreover, a copy of the survey can be found in the supplementary material.

The current dataset will be helpful for researchers, policymakers, educational managers and quality assurance specialists in the field. First, the data enable researcher to gain insights of students’ viewpoints on quality assurance as well as verify the research's results as data sharing is a practice that helps improve research integrity and transparency through enabling reproduction and peers' validation of the study's findings [11]. Second, the data will be beneficial to policymakers, institutional leaders, educational managers and quality assurance specialists to make decisions on quality assurance for their institutions and programmes. Data sharing offers valuable resources for scientific research and evidence-based policymaking, especially in developing countries, where scientific investments are limited and the quality of higher education is contentious [12]. Third, this dataset will contribute to knowledge management in the field of quality assurance. It is important to note that knowledge management is a vital aspect that datasets in social sciences and humanities should contribute to [13].

## 2 Experimental Design, Materials and Methods

The survey questionnaire was developed based on an international research project on internal quality assurance supported by UNESCO International Institute for Educational Planning (IIEP) in 2015-2016 [14]. Specifically, the back translation was used to translate the items from English into Vietnamese to ensure the reliability. The questionnaire was adjusted through discussing with five experts in quality assurance in Vietnam and pilot test with 76 students. The pilot test showed that the constructs achieved reliability when the Cronbach's Alpha values were greater than 0.8. In the official survey, the reliability of the questionnaire was again measured using Cronbach's Alpha value. Among 10 surveyed aspects, the Cronbach's Alpha value of each aspect was above 0.8 (Table 13), indicating a good level of reliability.

The questionnaire, which was adapted for the use in the context of Vietnam, consists of four parts and 60 close-ended questions. The first part includes four questions referring to personal information of respondents: year of study, gender, age and city of studying (Table 1). The second part having 29 questions focuses on quality policy and quality assurance model. This part is divided into six sub-sections including importance of education quality and quality policy, quality assurance handbook, institutional quality assurance body, purposes of quality assurance, and focus level of quality assurance (Table 2, Table 3, Table 4, Table 5, Table 6). The third part consisting of 21 questions refers to procedure and tools of quality assurance. This part is divided into three sub-sections including quality assurance instruments and processes, student support services, and instruments and processes for graduate employability (Table 7, Table 8, Table 9). The final part containing 10 questions highlights survey and evaluation. This part is divided in two sub-section including frequency of participating in evaluations and changes from evaluation results, (Table 10, Table 11).

**Table 1:** Description of the characteristics in the dataset.

Column	Data label	Explanation
A	Year	1; 2; 3; 4; 5; Others
B	Gender	Males; Female; I do not wish to say
C	Age	18; 19; 20; 21; 22; … [Participants fill in their age in number]
C	City	Hanoi; HCMC; Hue; Thai Nguyen; Vinh

**Table 2:** Importance of education quality and quality policy.

Column	Data label	Explanation
*Importance of education quality in the university's quality policy*		
E	Q2.1	0 [Do not know]; 1 [Not important]; 2 [Not really important]; 3 [Moderately important]; 4 [Important]; 5 [Very important]
*Do you know things related to quality policy or quality assurance policy in your university? (0 [Do not know]; 1 [No]; 2 [Yes])*		
F	Q2.2.1	Does your university have quality policy or quality assurance policy?
G	Q2.2.2	Is your university quality policy/ quality assurance policy clearly presented in the university strategies?
H	Q2.2.3	Do your university units/faculties have their own quality policy/ quality assurance policy?
I	Q2.2.4	Is the university quality policy/ quality assurance policy communicated to lecturers, staff and students?
J	Q2.2.5	Is the university quality policy/ quality assurance policy being developed?

**Table 3:** Quality assurance handbook (0 [Do not know]; 1 [No]; 2 [Yes]).

K	Q2.3.1	Does your university have a quality assurance handbook?
L	Q2.3.2	Does your university have documents describing quality assurance activities?
M	Q2.3.3	Do your university units/faculties have their own quality assurance handbook?
N	Q2.3.4	Is the university developing the quality assurance handbook?

**Table 4:** Unit in charge of quality assurance (0 [Do not know]; 1 [No]; 2 [Yes]).

O	Q2.4.1	Does your university have a unit responsible for quality assurance?
P	Q2.4.2	Does the quality assurance unit play an important role in controlling, ensuring and improving the education quality?
Q	Q2.4.3	Does the quality assurance unit collaborate with your faculty?
R	Q2.4.4	Have you ever contacted or worked with a staff of the quality assurance unit?
S	Q2.4.5	Have your lecturers ever told something about the role or activities of the quality assurance unit?

**Table 5:** Purposes of quality assurance (0 [Do not know]; 1 [Not important]; 2 [Not really important]; 3 [Moderately important]; 4 [Important]; 5 [Very important]).

T	Q2.5.1	Evaluating the education quality of the university
U	Q2.5.2	Improving teaching activities
V	Q2.5.3	Improving learning activities
W	Q2.5.4	Improving management activities
X	Q2.5.5	Improving support services
Y	Q2.5.6	Complying with government regulations
Z	Q2.5.7	Providing accountability to the government and society

**Table 6:** Focus level of quality assurance (0 [Do not know]; 1 [Not any]; 2 [Not much]; 3 [Moderate]; 4 [Quite a lot]; 5 [A lot]).

AA	Q2.6.1	Learning and teaching
AB	Q2.6.2	Graduate employability
AC	Q2.6.3	Research
AD	Q2.6.4	Management and governance
AE	Q2.6.5	Support services
AF	Q2.6.6	Facilities
AG	Q2.6.7	International cooperation

**Table 7:** Quality assurance instruments and processes (0 [Do not know]; 1 [No]; 2 [Yes]).

AH	Q3.1.1	Students’ evaluation of courses
AI	Q3.1.2	Students’ evaluation of programmes
AJ	Q3.1.3	Lecturers’ evaluation of programmes
AK	Q3.1.4	Programme monitoring based on statistical indicators
AL	Q3.1.5	Student progression monitoring
AM	Q3.1.6	Students’ workload assessment
AN	Q3.1.7	Student satisfaction survey

**Table 8:** Student support services (0 [Do not know]; 1 [No]; 2 [Yes]).

AO	Q3.2.1	Admission or registration for admission
AP	Q3.2.2	Academic consulting
AQ	Q3.2.3	Career counseling or job search
AR	Q3.2.4	Information technology facilities
AS	Q3.2.5	Library resources and learning materials
AT	Q3.2.6	Laboratories and practice rooms
AU	Q3.2.7	Health and medical consulting
AV	Q3.2.8	Accommodation consulting

**Table 9:** Instruments and processes for graduate employability (0 [Do not know]; 1 [No]; 2 [Yes]).

AW	Q3.3.1	Graduate tracer research
AX	Q3.3.2	Employer survey
AY	Q3.3.3	Curriculum development involving the professions/employers
AZ	Q3.3.4	Curriculum review involving the relevant professions
BA	Q3.3.5	Curriculum review involving alumni
BB	Q3.3.6	Monitoring the quality of internships

**Table 10:** Frequency participating in surveys (0 [Do not know]; 1 [Never]; 2 [Rarely]; 3 [Sometimes]; 4 [Often]; 5 [Always]).

BC	Q4.1.1	Students’ evaluation of teachers
BD	Q4.1.2	Students’ evaluation of subjects
BE	Q4.1.3	Students’ evaluation of courses
BF	Q4.1.4	Students’ evaluation of programmes
BG	Q4.1.5	Students’ evaluation of support services
BH	Q4.1.6	Students’ evaluation of facilities

**Table 11:** Positive changes from evaluation results (0 [Do not know]; 1 [No change]; 2 [Change a little]; 3 [Change some]; 4 [Change quite a lot]; 5 [Change a lot]).

BI	Q4.2.1	Positive change in teaching performance
BJ	Q4.2.2	Positive change in support services
BK	Q4.2.3	Positive change in testing and assessment
BL	Q4.2.4	Positive change in facilities

**Table 12:** Demographic characteristics of the participants (N = 1323).

Variable	Frequency	Percent
Year		
1	255	19.3
2	155	11.7
3	456	34.5
4	443	33.5
5	9	0.7
Others	5	0.3
Gender		
Male	179	13.5
Female	1138	86.0
I do not wish to say	6	0.5
Age		
18	223	16.9
19	130	9.8
20	344	26.0
21	385	29.1
22	193	14.6
23	22	1.7
24	8	0.6
25	3	0.2
28	2	0.2
31	2	0.2
32	2	0.2
33	3	0.2
34	1	0.1
35	1	0.1
37	1	0.1
39	2	0.2
45	1	0.1
City	223	16.9
HCMC	553	41.8
Hanoi	263	19.9
Hue	164	12.4
Thai Nguyen	181	13.7
Vinh	162	12.2

**Table 13:** Results of Cronbach's Alpha for surveyed aspects.

No.	Surveyed aspects	No. of items	Cronbach's Alpha
1	Importance of education quality and quality policy (Q2.2.1 - Q2.2.5)	5	0.878
2	Quality assurance handbook (Q2.3.1 - Q2.3.4)	4	0.836
3	Unit in charge of quality assurance (Q2.4.1 - Q2.4.5)	5	0.912
4	Purposes of quality assurance (Q2.5.1 - Q2.5.7)	7	0.985
5	Focus level of quality assurance (Q.2.6.1 - Q2.6.7)	7	0.964
6	Quality assurance instruments and processes (Q.3.1.1 - Q3.1.7)	7	0.894
7	Student support services (Q.3.2.1 - Q3.2.8)	8	0.902
8	Instruments and processes for graduate employability (Q.3.3.1 - Q3.3.6)	6	0.931
9	Frequency of participating in surveys (Q.4.1.1 - Q4.1.6)	6	0.948
10	Positive changes from evaluation results (Q.4.2.1 - Q4.2.4)	4	0.959

A Google Form was created to add questions in Vietnamese from the developed survey. To maximise the diversity of respondents, the researchers selected students studying in five cities representing all main regions of Vietnam including Thai Nguyen (northeastern part), Hanoi (capital city), Vinh (north central coast), Hue (near south central coast) and Ho Chi Minh City (southeastern part). The total population of this study was over one million students. Convenience sampling was used to select students from universities located in these cities. Specifically, the questionnaire was shared among the students in these cities with the help of their university's managers, teachers, student advisors, and their friends as well as through social networks. The informed consent was provided to potential participants in the email directly sent to them (or forwarded to them). From July to September 2020, the questionnaire received 1471 responses. Unreliable data was eliminated by scanning responses for identical responses, missing more than a third of the scale/sub-scale, and missing more than two items of personal information. After that, an adjustment was made to remove outliers. The number of valid responses remained at 1323. Because of the convenience sampling procedures, this dataset does not represent the entire population of students currently studying at higher education institutions in Vietnam. The detail of the survey questionnaire and the responses are provided in the supplementary files.

## Ethics Statements

Ethical review and approval was not required for the study on human participants in accordance with the local legislation and institutional requirements. Informed consent was obtained from respondents prior to completion of the online survey.

## CRediT authorship contribution statement

**Hien Thi Thu Ta:** Conceptualization, Methodology, Writing – original draft. **Cuong Huu Nguyen:** Supervision, Writing – review & editing. **Hung Thai Le:** Data curation, Validation. **Nhung Thi Tuyet Pham:** Investigation, Writing – original draft. **Huong Thi Pham:** Software, Validation. **Nhung Thi Trinh:** Investigation, Validation.

## Declaration of Competing Interest

The authors declare that they have no known competing financial interests or personal relationships that could have appeared to influence the work reported in this paper.

## Data Availability

Survey dataset on student perceptions and experiences of quality assurance in Vietnamese universities (Original data) (Mendeley Data).
